# Engineering of fructose-6-phosphate aldolase for one-carbon conversion to mannitol in a designed biotransformation system

**DOI:** 10.1016/j.synbio.2026.04.009

**Published:** 2026-06-05

**Authors:** Dandan Wang, Qianzhen Dong, Peng Chen, Yan Zeng, Yinlu Liu, Yuanxia Sun, Jianxin Tan, Jiangang Yang

**Affiliations:** a College of Food Science and Technology, Hebei Agricultural University, Baoding, 071001, China; b Tianjin Institute of Industrial Biotechnology, Chinese Academy of Sciences, Tianjin, 300308, China; c Key Laboratory of Engineering Biology for Low-carbon Manufacturing, Tianjin, 300308, China

**Keywords:** Fructose-6-phosphate aldolase, One-carbon, Reaction mechanism, Multienzyme system, Mannitol

## Abstract

Developing artificial synthetic pathways for converting one-carbon compounds into value-added chemicals represents a promising strategy for carbon-neutral manufacturing. Enzymatic C–C bond formation plays a central role in carbon-chain extension and structural diversification. Fructose-6-phosphate aldolase (FSA) has been employed in *in vitro* multienzyme systems for producing starch and sugars from methanol. However, its atomistic catalytic mechanism has remained unclear, limiting rational enzyme engineering. Here, we elucidate the aldol reaction mechanism between dihydroxyacetone (DHA) and glyceraldehyde-3-phosphate (GALP) catalyzed by FSA using QM/MM calculations, identifying the Schiff-base/iminium formation step as the rate-limiting step in the aldol condensation. Guided by this mechanism, we engineered FSA variants with a 34-fold increase in catalytic efficiency compared with the wildtype. We further integrated the engineered FSA into a designed *in vitro* cascade converting methanol to mannitol, achieving a high yield of 88%. Collectively, these mechanistic insights and improved biocatalysts expand the toolkit for green enzymatic C–C bond formation and one-carbon utilization.

## 1 Introduction

Bioconversion of one-carbon resources, such as CO_2_, methanol, and formaldehyde, into high-value products is an effective strategy for achieving carbon-neutral manufacturing [[1], [2], [3]]. To date, seven natural CO_2_ fixation pathways have been discovered [4]; however, they involve complex reaction steps, high ATP consumption, or low conversion rates [5,6]. Therefore, many synthetic pathways have been constructed with the aid of metabolic pathway design and enzyme engineering [7,8]. Enzymatic carbon–carbon bond formation reactions allow for modular assembly of functionalized carbon skeletons, providing programmable access to diverse long-chain products [9]. Particularly, aldol reactions are powerful due to their high selectivity and adaptability [10]. In recent years, the chem-enzymatic platforms ASAP [11] and ACSP [12] have demonstrated success in converting carbon dioxide into starch, hexoses, and sucrose. In these pathways, carbon condensation of formaldehyde to dihydroxyacetone (DHA) and sequential conversion of DHA to fructose-6-phosphate (F6P), the key precursor for synthesis of starch and hexoses, were achieved with the aid of formolase [13] and aldolases. Therefore, aldolases with high catalytic efficiency and substrate specificity are essential for efficient conversion of low-carbon compounds into high-order sugars in this *in vitro* biotransformation platform.

In cells, the metabolic network that converts DHA to F6P relies primarily on fructose-bisphosphate aldolase (FBA), which catalyzes the condensation of dihydroxyacetone phosphate (DHAP) and glyceraldehyde-3-phosphate (GALP) to fructose-1,6-bisphosphate (F1,6BP) [14], followed by fructose-1,6-bisphosphatase (FBPase) converting F1,6BP to F6P [15]. Recently, fructose-6-phosphate aldolase (FSA), which catalyzes the condensation of DHA and GALP to F6P [16], has attracted increasing attention because it enables a shorter route with lower ATP consumption than the FBA route [12]. This enzyme is particularly useful in the designed ASAP and ACSP system for carbohydrates production from CO_2_. Several crystal structures of FSA have been solved, and key catalytic residues of Lys85 and Tyr131 have also been identified [[17], [18], [19]]. However, a complete atomistic catalytic cycle (including the Michaelis complex and transition state) is still lacking, constraining rational engineering for improved activity and substrate specificity. Computational chemistry methods, such as QM and QM/MM, provide atomistic views of key intermediates and transition states [20], enabling the identification of rate-determining events [21], and, by furnishing quantitative transition-state models, they have also become key tools for de novo design of new enzymes and even new reactions [22,23].

The reaction mechanism of class I aldolase is often conceptualized as a two-phase process comprising a donor-activation phase (Lys-mediated Schiff-base formation) followed by an acceptor addition and product release phase, in which the nucleophilic enamine attacks the acceptor carbonyl to forge the C–C bond and the resulting imine is hydrolyzed to regenerate the free enzyme [24]. To date, structure-guided pocket remodeling of FSA from *E. coli* (EcoFSA) has been conducted to extensively broaden the substrate scope for both nucleophile donors and aldehyde acceptors [25]. For example, enzyme engineering of the donor-scope pocket produced beneficial mutants, such as A129S [26], L107/A129/A165 [27], and D6X variants [28], which exhibited enhanced affinity to DHA, glycolaldehyde or aliphatic ketones/aldehydes as viable donors relative to the wild type. On the acceptor side, variants such as A129S/A165G [29] and higher-order combinations (e.g., A129S/R134X/A165G/S166G) [30] deliver high activity and stringent stereocontrol with N–Cbz–aminoaldehydes and other bulky electrophiles. However, few attempts have been focused on improving the native DHA and GALP condensation reaction, which is directly relevant to low-carbon carbohydrate biomanufacturing.

Here, we report the catalytic cycle of aldol reaction between DHA and GALP in EcoFSA using QM/MM method. Step-by-step free-energy profiles and transition-state structures were obtained for each elementary event. We identify Schiff-base/iminium formation during donor activation as the primary rate-limiting step for aldol condensation. Guided by this mechanism, we further performed structure-guided engineering of the donor and acceptor pockets and identified a beneficial mutant, FSA-M3(A129S/S30T/K168R), exhibiting a 34-fold increase in catalytic efficiency relative to wild-type FSA. Finally, we designed and constructed an *in vitro* biotransformation system converting methanol and formate to mannitol with an 88% conversion yield, presenting the first report for synthesis of mannitol from a one-carbon source.

## 2 Materials and methods

### 2.1 Materials, strains and culture conditions

DHA and GALP were purchased from Meryer Chemical Group (Shanghai, China) and Sigma-Aldrich (St. Louis, MO, USA), respectively. Nicotinamide adenine dinucleotide (NAD+) and isopropyl β-d-1-thiogalactopyranoside (IPTG) was purchased from Solarbio (Beijing, China). *E. coli* DH5α was used for routine cloning, and *E. coli* BL21(DE3) was used for protein expression. Strains were cultured in Luria–Bertani (LB) medium at 37 °C with shaking at 200 rpm. Where appropriate, ampicillin was added at 100 μg/mL. For protein expression, overnight seed cultures were inoculated (typically 1:100, v/v) into fresh LB medium and grown to an OD600 of 0.6–0.8, then induced with 0.5 mM IPTG and incubated at 16 °C for 18–20 h.

### 2.2 Plasmid construction and mutagenesis

The EcoFSA gene (Uniprot: P78055) was amplified from the *E. coli* genome and cloned into the pET-21a plasmid to generate an N-terminal 6 × His-tagged EcoFSA. Site-directed mutagenesis and combinatorial mutagenesis were performed using PCR-based methods, and all mutations were verified by Sanger sequencing (Genewiz, Suzhou, China). For mutations at Asn28, Thr109, Ala129, Ser30, Ser166, and Lys168, each variant was generated individually by site-directed mutagenesis using custom-designed mutagenic primer pairs. Briefly, primers carrying the desired nucleotide substitutions were designed to introduce the target amino-acid change at the center of the primer. Mutagenesis PCR was performed using the parental plasmid as the template, followed by *Dpn*I digestion to remove parental DNA. The amplified products were purified and transformed into *E. coli* DH5α for plasmid propagation. Correct genotypes were confirmed by Sanger sequencing of the full FSA coding region. For multi-site variants, mutations were introduced iteratively by using a sequence-verified single-mutant plasmid as the template for the next round of mutagenesis.

### 2.3 Protein expression and purification

For rapid screening, cell pellets from 50 mL cultures were prepared by centrifugation (5000×*g*, 15 min) and resuspended in TEA buffer (50 mM triethanolamine, pH 7.5, 150 mM NaCl). Cells were disrupted by sonication on ice, and the crude lysate was heat-treated at 60 °C for 30 min to precipitate most host proteins. After centrifugation, the supernatant was collected as ‘crude enzyme’ for activity assays; the quality of crude preparations was assessed by SDS–PAGE.

For purification, cell pellets were resuspended in binding buffer (50 mM TEA, pH 7.5, 500 mM NaCl, 20 mM imidazole) and lysed by sonication at 4 °C. After centrifugation (13,000×*g*, 30 min), the supernatant was loaded onto a Ni2+–NTA column pre-equilibrated with binding buffer. The column was washed with 10 column volumes of binding buffer and the 6 × His-tagged EcoFSA was eluted with elution buffer (50 mM TEA, pH 7.5, 500 mM NaCl, 500 mM imidazole). Eluted fractions were concentrated and stored in buffer (50 mM TEA, pH 7.5, 150 mM NaCl). Glycerol was added to 5% (v/v), and proteins were stored at −20 °C. Protein purity was assessed by SDS-PAGE, and protein concentration was determined using the BCA assay with bovine serum albumin as the standard.

### 2.4 EcoFSA activity assay using DHA and GALP

EcoFSA activity was quantified by F6P formation and enzymatic dephosphorylation to fructose. Standard reactions (50 μL) contained 20 μg EcoFSA-WT (1–2 μg for A129S and variants), 50 mM HEPES (pH 7.5), 20 mM DHA, and 10 mM GALP. The reactions were incubated at 30 °C for 10 min and quenched by boiling for 10 min. After brief centrifugation, 25 μL heat-treated fructose-6-phosphate phosphatase (F6PP) was added and incubated at 55 °C for 120 min to completely convert F6P to fructose, followed by boiling for 10 min. To determine the kinetic parameters of GALP, the concentration of GALP was varied from 0 to 8 mM at a constant DHA concentration of 100 mM. Fructose was quantified by HPLC. One unit (U) of activity was defined as the amount of enzyme producing 1 μmol fructose per minute under the assay conditions. The enzyme activity method differed from the reported method [29], in which the generated F6P was quantified through a coupled assay using phosphoglucose isomerase and glucose-6-phosphate dehydrogenase, accompanied by NADPH generation. The NADPH concentration was measured by absorbance at 340 nm using a plate reader.

### 2.5 *In vitro* cascade for mannitol production from DHA or methanol

For the C6-module validation using DHA as the input, DhaK from *Pichia pastoris* (PpDhaK, Uniprot id: O74192), triosephosphate isomerase (TPI) from *E. coli* (EcTPI, Uniprot id: P0A858), EcoFSA variants, an F6PP mutant from *Thermotoga* sp. 38H (F6PP3, Uniprot id: UPI0013E9B491) [31], mannitol dehydrogenase (MDH) from *Leuconostoc pseudomesenteroides* (LpMDH, Uniprot id: Q83VI5), and formate dehydrogenase (FDH) from *Mycobacterium vaccae* (MvFDH, Uniprot id: Q93GV1) were used. For the upstream C1-to-C3 module, alcohol oxidase (AOX) from *P. pastoris* (PpAOX, Uniprot id: P04842), catalase (Cat) from *Bacillus subtilis* (BsCat, Uniprot id: P26901), and a formolase mutant FLS-M3 were used [11].

Unless otherwise stated, reactions (100 μL) contained DHA (200 mM), HEPES buffer (200 mM, pH 7.5), ATP (2 mM), MgCl_2_ (30 mM), sodium formate (150 mM), NAD^+^ (1 mM), PpDhaK (0.12 mg/mL), polyphosphate kinase (PPK; 0.22 mg/mL), EcTPI (0.16 mg/mL), EcoFSA variant (0.15 mg/mL), F6PP (0.40 mg/mL), LpMDH (0.60 mg/mL), and MvFDH (1.20 mg/mL). Reactions were incubated at 30 °C for 6 h and terminated by boiling for 10 min prior to HPLC quantification of mannitol. For module optimization experiments, the concentrations of EcoFSA, F6PP, MDH, and polyphosphate concentration were varied as indicated in the figure legends, while all other components were held constant. One-pot methanol-to-mannitol was performed in two-stage reactions: in stage 1 (C1-to-C3), methanol (200 mM) was incubated in 200 mM HEPES (pH 7.5) containing PpAOX (0.75 mg/mL), BsCat (0.42 mg/mL), and FLS-M3 (5 mg/mL) at 30 °C. Samples were taken at different reaction time points and heat-quenched in tightly capped microcentrifuge tubes (boiling-water bath, 10 min). Tubes were cooled on ice and then centrifuged before opening for methanol analysis. The control experiment was designed with the same concentration of methanol in buffer without other components. After ∼4 h, stage 2 (C3-to-C6) was initiated by adding the enzyme components DhaK, PPK, TPI, EcoFSA, F6PP, MDH, and FDH. For overall methanol-to-mannitol cascade, the conversion yield (%) was calculated on a carbon-molar basis, this gives *Y*_C_ (%)= (6 × [mannitol])/[methanol]_initial_ × 100, consistent with the overall stoichiometry of 6 mol methanol per mol mannitol. For DHA-to-mannitol assays, conversion yield (%) was calculated relative to *Y*_C_ (%)= (6 × [mannitol])/(3 × [DHA]_initial_) × 100.

### 2.6 Determination of FAD loading of AOX

The FAD (Flavin adenine dinucleotide) loading of the AOX preparation used in this study was evaluated spectrometrically. Free FAD standards were first analyzed by UV–visible scanning from 300 to 600 nm in a quartz cuvette, which showed the characteristic flavin absorption bands around 375 and 450 nm (Shimadzu, UV-1900i). Because no appreciable absorbance of free FAD was observed at 562 nm, AOX protein concentration was determined independently by the BCA assay. For FAD quantification, a standard curve was established at 450 nm in a 96-well microplate using FAD concentrations of 0, 20, 40, 60, 80, and 100 μM in a volume of 200 μL per well (BioTek, Synergy LX). AOX samples were adjusted to 2, 4, and 6 mg/mL according to the BCA-measured protein concentration, heat-denatured to release bound FAD, and the released FAD was quantified directly at 450 nm without further dilution. FAD occupancy was calculated by comparing the measured FAD concentration with the theoretical value expected for fully loaded AOX, assuming one FAD per 74-kDa AOX monomer.

### 2.7 MD simulation and trajectory analysis

To generate initial EcoFSA complexes, the decameric crystal structure of *E. coli* fructose-6-phosphate aldolase (EcoFSA; PDB: 1L6W) was used as the receptor template [17]. The covalent modification of the catalytic Lys85 reported in the crystal structure (Lys85 carbinolamine adduct originating from glyceraldehyde in the crystallization mixture) and all non-protein heteroatoms were removed prior to modeling. Two receptor models were prepared depending on the simulation setup: (i) a single protomer (one subunit) extracted from the decamer for monomeric simulations and docking-based pose generation; and (ii) a pentameric ring constructed by retaining chains A–E from the 1L6W biological assembly to preserve native inter-subunit contacts within one ring. For generation of initial EcoFSA complexes, one protomer was extracted from the decameric crystal structure (PDB: 1L6W) and the covalently bound ligand was removed. The substrates DHA and GALP were docked into the active-site region using AutoDock Vina 1.2.5 [32] to obtain starting conformations for subsequent MD or QM/MM simulations. Docking boxes were centered on the Nζ atom of Lys85 for DHA docking and on Arg134 (guanidinium center) for GALP docking, with a box size of 24 × 24 × 24 Å^3^.

All-atom MD simulations were performed using GROMACS [33] patched with the Colvars module [34]. The protein was parameterized using the CHARMM36m force field [35], and DHA/GALP parameters were assigned using the CHARMM General Force Field (CGenFF) [36]. The systems were solvated in a cubic TIP3P [37] water box with at least 15 Å padding from the protein surface, neutralized, and supplemented with NaCl to 150 mM. Energy minimization was carried out using 10,000 steps of steepest descent. Equilibration was performed in the NVT ensemble for 125 ps with positional restraints on heavy atoms, followed by NPT equilibration (1 bar) as needed. Production simulations were run at 300 K and 1 bar using the Nosé–Hoover thermostat [38,39] and Parrinello–Rahman barostat [40]. Long-range electrostatics were treated with PME [41,42]. All bonds involving hydrogen were constrained using the LINCS algorithm [43], allowing a 2-fs integration time step. Nonbonded interactions were treated using the Verlet cutoff scheme. Short-range electrostatic and van der Waals interactions were truncated at 12 Å. A force-switching function was applied to the Lennard–Jones interactions from 10 Å to 12 Å. RMSF values were calculated from the 3 × 500 ns trajectories (heavy atoms of each residue) using *gmx rmsf* after least-squares fitting to the starting structure.

### 2.8 Tunnel analysis

Substrate tunnels in the decameric EcoFSA structure (PDB: 1L6W) were analyzed using the CAVER 3.0 PyMOL plugin [44]. The covalently bound ligand was used to define the starting point for tunnel calculation. The VMD (v1.9.4) [45] and PyMOL (v3.1, Schrödinger, LLC) were used for visualization and trajectory analysis.

### 2.9 QM/MM MD simulation and free-energy calculation

QM/MM simulations were performed with NAMD 3.0 [46,47] interfaced to ORCA 6.0 [48]. A 0.5 fs time step was used for QM/MM MD. To balance computational accuracy and cost, the QM region was treated with the tight-binding GFN2-xTB method [49] under electrostatic embedding; covalent QM/MM boundaries were treated using the link-atom approach.

For EcoFSA-WT donor-activation simulations (through enamine formation), the QM region included the side chains of Asp6, Asn28, Gln59, Lys85, Thr109, Ala129, and Tyr131, as well as the DHA donor (79 atoms; net charge −1). For water-mediated dehydration (*d*_water_ and *d*_4_), the nearest catalytic water molecule was additionally included (82 atoms). For acceptor addition and product release (*d*_6_–*d*_10_), the entire GALP molecule was included (94 atoms; net charge −3). For EcoFSA-A129S, Ala129 was replaced by Ser (80 atoms; net charge −1 for the corresponding donor-activation QM region).

The potential of mean forces (PMFs) of donor activation, acceptor addition and system regeneration in QM/MM MD were all calculated by the well-tempered variant of *meta*-eABF (WTM-eABF) [50,51] in the Colvars module. Each trajectory was propagated for at least 50 ps.

WTM-eABF method was also applied to the process of GALP recruitment for EcoFSA-WT and EcoFSA-M3 assemblies at MM level. All free-energy profiles reported in this study were obtained over three independent WTM-eABF simulations.

### 2.10 Analytical methods

The HPLC system (Agilent 1260) equipped with a refractive index detector (Agilent G1362A) and cation-exchange columns was used for HPLC analysis. For the Sugar-Pak™ column, deionized water was used as the mobile phase at a flow rate of 0.4 mL/min, and the column temperature was controlled at 80 °C. For the Bio-Rad Aminex HPX-87H column, a 5 mM H_2_SO_4_ mobile phase at 0.5 mL/min was used, and the column temperature was controlled at 55 °C. For post-processing of the data from HPLC analysis, the peak area for the target product was obtained by automatic and manual integration and then calculated using the calibration curve for sugars.

## 3 Results and discussions

### 3.1 The underlying reaction mechanism of donor activation in FSA

The FSA from *E.coli* catalyzes the reversible carbon–carbon bond formation between DHA and GALP to yield F6P. In the aldol reaction, donor activation denotes the sequence of elementary steps that convert DHA into a covalently bound, nucleophilically “primed” species (iminium/enamine) prior to C–C bond formation with the acceptor. This activation proceeds by a canonical Schiff-base strategy without requiring divalent cations, in which a catalytic lysine forms a covalent adduct with the donor, followed by dehydration and α-deprotonation to generate an enamine nucleophile.

**Fig. 1:**
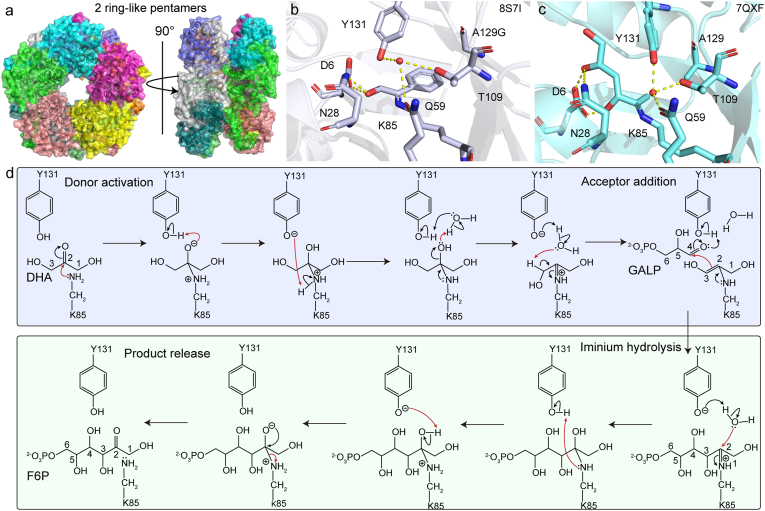
Decameric architecture and reaction mechanism of EcoFSA. a) Surface representation of the FSA decamer. b) Close-up of the active site in an engineered FSA structure (PDB 8S7I). The key residues (Asp6, Asn28, Gln59, Thr109, Gly129(A129G) and Tyr131) surrounding the Lys85 are shown with hydrogen-bonding interactions indicated by dashed lines. c) Close-up of the active site in an engineered FSA structure (PDB 7QXF). The surrounding residues Asp6, Asn28, Gln59, Thr109, Ala129 and Tyr131 are shown. d) Schematic of the proposed reaction mechanism of condensation of DHA and GALP to yield F6P, catalyzed by EcoFSA, which could be partitioned into donor activation, acceptor addition, iminium hydrolysis and product release. Red curved arrows depict the electron-pair flow associated with the reaction coordinates (collective variables) used in the QM/MM PMF calculations. Abbreviations: DHA, dihydroxyacetone; GALP, glyceraldehyde-3-phosphate; F6P, fructose-6-phosphate.

Structurally, FSA assembles as a dimer of ring-like pentamers, and each monomeric subunit adopts a (β/α)_8_ TIM-barrel fold with the active site located at the barrel entrance. The overall oligomeric architecture involves helix swapping between neighboring subunits (Fig. 1a). Notably, several EcoFSA crystal structures have shown a covalent Lys85-linked carbinolamine/hemiaminal adduct (Fig. 1b and c), providing direct structural evidence that donor activation via Schiff-base chemistry precedes C–C bond formation.

Among active-site residues, Lys85 is the covalent catalyst, and mutating this residue abolishes activity. Tyr131 is positioned as the dominant proton shuttle in FSA and has been repeatedly implicated in facilitating both dehydration and subsequent proton-transfer events during turnover. Beyond the Lys85/Tyr131 dyad, residues Asp6, Asn28, Gln59, Thr109 and Ala129 cluster around the donor-binding pocket (Fig. 1b and c) and are frequently targeted in protein engineering because they modulate substrate positioning and hydrogen-bonding patterns. In particular, residues Asp6 and Asn28 form direct contacts with donor-derived intermediates and are recognized as determinants of donor recognition and hot spots for enzyme engineering to expand donor scope [28]. In addition, Gln59 and Thr109, together with Tyr131, organize a hydrogen-bond network capable of stabilizing a conserved catalytic water molecule [52]. Accordingly, Asp6, Asn28, Gln59, Thr109, Lys85, and Tyr131 were included for QM simulation. To explicitly examine the contribution of the donor-pocket mutation, Ala129 (A129S in our variant) was also treated quantum mechanically.

**Fig. 2:**
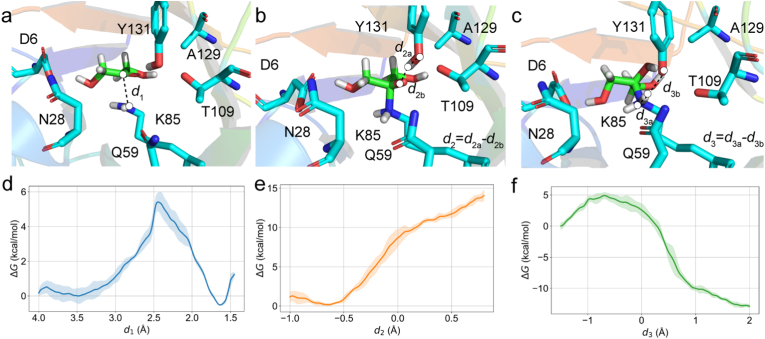
QM/MM free-energy profiles for donor activation in EcoFSA. a) Definition of the reaction coordinate *d*_1_, the distance between Lys85 Nζ and the donor carbonyl carbon (C2 of DHA), describing the nucleophilic attack step in the donor pocket. b) Definition of the proton-transfer coordinate *d*_2_ = *d*_2a_ − *d*_2b_, where *d*_2a_ and *d*_2b_ are distances from the transferring proton to the Tyr131 phenolic oxygen and the donor oxyanion (O2), respectively, capturing Tyr131-to-donor protonation that neutralizes the zwitterionic carbinolamine. c) Definition of the proton-transfer coordinate *d*_3_ = *d*_3a_ − *d*_3b_, where *d*_3a_ monitors the distance between the transferable proton and the Nζ atom of Lys85, and *d*_3b_ monitors the distance between that proton and the Tyr131 oxygen, describing proton exchange that regenerates Tyr131 and stabilizes the Lys85–donor adduct. d, e, f) Corresponding relevant free-energy profiles generated from QM/MM simulations for nucleophilic addition (*d*_1_), Tyr131-mediated protonation of the carbinolamine oxygen (*d*_2_) and regeneration of Tyr131 (*d*_3_). Data are presented as mean values ± the standard error inferred from three independent runs, shaded bands indicate the standard error.

We initiated the simulations from the monomer of the decameric FSA structure (PDB 1L6W) and generated an apo starting model by removing the covalent glyceraldehyde adduct present in the crystal structure. Based on the experimentally verified depressed p*K*_a_ of the catalytic lysine (p*K*_a_ = 5.5) [52], Lys85 was modeled in its neutral (deprotonated) nucleophilic state prior to donor addition. The donor-activation process started with a nucleophilic attack by the Nζ atom of Lys85 on the DHA carbonyl carbon. The reaction coordinate (RC) *d*_1_ was defined as the distance between Nζ atom of Lys85 and the carbonyl carbon (C2) of DHA, describing the nucleophilic attack to DHA (Fig. 2a). The resulting potential of mean force (PMF) indicates that the covalent addition is thermodynamically feasible, with a calculated free-energy barrier of 5.7 ± 0.5 kcal/mol (Fig. 2d). Along this coordinate, the nucleophilic attack converts the planar C=O group of DHA into a tetrahedral center and yields a Lys85-linked carbinolamine intermediate. The former carbonyl oxygen carries a negatively charged oxyanion that strongly attracts the phenolic proton of Tyr131, suggesting early geometric pre-organization for the subsequent proton-transfer step (Supplementary Fig. 1).

In the following step, the zwitterionic carbinolamine should be protonated at O2 to generate a neutral carbinolamine moiety that can undergo dehydration. In our simulations, this protonation is mediated by Tyr131. We defined *d*_2_ as a difference-of-distances coordinate (*d*_2_ = *d*_2a_ – *d*_2b_) to capture proton transfer from the Tyr131 phenolic oxygen to O2 of the donor fragment, where *d*_2a_ and *d*_2b_ denote the distances between the transferring proton and the phenolic oxygen of Tyr131 and the donor oxyanion, respectively (Fig. 2b). Negative values of *d*_2_ correspond to the proton being closer to Tyr131 (reactant), whereas positive values indicate that it is closer to the donor oxygen O2 (product) (Fig. 2e).

To neutralize the carbinolamine, the proton initially retained on Lys85 after nucleophilic attack is transferred from Lys85 Nζ to the Tyr131 tyrosinate, thereby regenerating protonated Tyr131 and leaving the Lys85–C2 adduct in neutral and stable state. We described this exchange by the RC *d*_3_ = *d*_3a_ − *d*_3b_ (Fig. 2c). Here, *d*_3a_ monitors the distance between the transferable proton and Lys85 Nζ, and *d*_3b_ monitors the distance between that proton and the Tyr131 oxygen. The proton transfer proceeds with a small barrier (4.9 ± 0.5 kcal/mol) and is overall downhill (−12.9 ± 0.3 kcal/mol) on the PMF, indicating that the process is accessible and thermodynamically favorable (Fig. 2f). Importantly, this step restores Tyr131 as a proton donor for the ensuing dehydration while removing excess positive character from the lysine nitrogen. Over these two proton-transfer steps following the initial nucleophilic attack, the Nζ(Lys85)–C2(donor) bond length decreases from ∼1.55 Å to ∼1.45 Å (Supplementary Figs. 2 and 3). It was in good agreement with reported C–N bond lengths for protonated and neutral carbinolamine intermediates in pyridoxal 5′-phosphate-dependent enzymes (∼1.53 Å) [53] and for small-molecule hemiaminals in crystallographic studies (1.46–1.47 Å) [54].

Dehydration of the formed neutral carbinolamine is the decisive transformation that installs the C=N iminium (Schiff-base) linkage. It converted a relatively unactivated tetrahedral adduct into a conjugated “electron-sink” intermediate, and thereby enables α-deprotonation to the nucleophilic enamine, further gating donor activation and subsequent C–C bond formation. For EcoFSA and its transaldolase homologs, available structural and functional evidence has been interpreted as most consistent with a catalytic-water-mediated dehydration, in which a conserved water coordinated by the Q59–T109–Y131 network participates in acid–base chemistry, rather than a mechanism that relies exclusively on direct proton donation from Tyr131. Stellmacher et al. [52] identified this conserved water and showed that swapping residues that organize it between FSA and TalB interconverts aldolase and transaldolase activity, implicating the water network as a key determinant of reactivity. Consistent with this view, Cornelius et al. [18] modeled the carbinolamine-to-iminium conversion as proton transfer via an active-site water coordinated by the same triad, while also noting conformations compatible with direct Tyr131 protonation. Accordingly, we focus on the Tyr131–water–substrate proton-relay pathway as the primary route in wild-type EcoFSA, while treating a purely Tyr131-mediated dehydration as a plausible alternative.

**Fig. 3:**
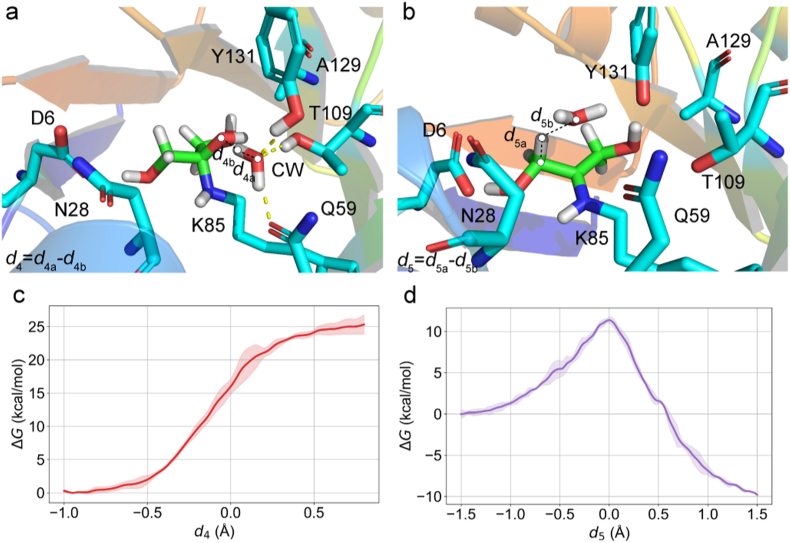
QM/MM free-energy profiles for catalytic-water-mediated dehydration of the Lys85-linked carbinolamine and subsequent α-deprotonation in FSA. a) Definition of the proton-transfer coordinate *d*_4_ = *d*_4a_ − *d*_4b_, where *d*_4a_ and *d*_4b_ are distances from the transferring proton to the catalytic water oxygen and the carbinolamine hydroxyl oxygen (leaving group), respectively, describing CW-assisted protonation and water loss that converts the Lys85-bound carbinolamine into the iminium (Schiff-base) intermediate. b) Definition of the α-deprotonation coordinate *d*_5_ = *d*_5a_ − *d*_5b_, where *d*_5a_ captures C3–H bond cleavage on the donor fragment and *d*_5b_ captures proton transfer to the oxygen atom of the leaving-group water (LG), describing conversion of the iminium into the nucleophilic enamine intermediate. c, d) Corresponding relevant free-energy profiles generated from QM/MM simulations for CW-mediated dehydration (*d*_4_) and enamine formation via α-deprotonation (*d*_5_), revealing dehydration as the dominant kinetic bottleneck within the donor-activation module, whereas enamine formation is comparatively accessible and thermodynamically favored. Data are presented as mean values ± the standard error inferred from three independent runs; shaded bands indicate the standard error.

In our simulations, the water molecule nearest to the catalytic site was selected to mediate the reaction. The approach of the catalytic water was described by the collective variable (CV) *d*_water_, defined as the distance between the CW oxygen atom and the center of mass (COM) of the side-chain oxygen atoms of Gln59, Thr109 and Tyr131 (Supplementary Fig. 4a). The potential of mean force along *d*_water_ shows a pronounced minimum at ∼1.8 Å, where the water simultaneously forms hydrogen bonds with Q59, T109 and Y131, indicating that the Q59–T109–Y131 triad forms a well-defined, strongly bound CW site. (Supplementary Fig. 4b). After placing the CW in the Q59–T109–Y131 pocket, we next examined the water-mediated dehydration of the Lys85-bound carbinolamine to the iminium intermediate (Fig. 3a). The proton transfer from the CW to the carbinolamine hydroxyl was described by an antisymmetric distance RC *d*_4_ = *d*_4a_ - *d*_4b_ (Fig. 3b), and the resulting free-energy profile along *d*_4_ shows a broad maximum of 24 kcal/mol, thereby identifying dehydration as the rate-limiting step within the donor-activation module under our simulation conditions (Fig. 3d). Notably, this discovery is consistent with the previous reported QM/MM study of DHAP-dependent class I Schiff-base aldolase (Δ*G* = 20.6 kcal/mol) [55]. In our simulations, the proton transfer was accompanied by the proton transfer from the sidechain of Tyr131 to CW and cleavage of the donor C–O to create a leaving group (LG) (Supplementary Movie 1). Along the same QM/MM MD trajectories, the Lys85–C2 bond distance *d*_1_ shortens from single-bond-like values in the carbinolamine region to double-bond-like values in the iminium region (Fig. 3a, Supplementary Fig. 5b), consistent with conversion of a neutral carbinolamine into a cationic iminium species.

Finally, the iminium intermediate needs to be converted into the enamine by α-deprotonation of the donor fragment. We then analyzed the reversible conversion of the iminium into the nucleophilic enamine intermediate. This α-deprotonation process was quantified using the *d*_5_ = *d*_5a_ − *d*_5b_, where *d*_5a_ captures C3–H bond breaking and *d*_5b_ captures proton transfer to the oxygen atom of the LG. (Fig. 3b). The result exhibits a moderate barrier of 10 kcal/mol and a downhill free-energy change toward the enamine region, accompanied by proton transfer from LG to the Tyr131 tyrosinate (Fig. 3d, Supplementary Movie 2), indicating that enamine formation is comparatively facile and thermodynamically favored. The structural evolution along these trajectories corroborates this assignment: during the enamine-forming step, the Nζ–C2 distance (*d*_1_) increases while the adjacent C2–C3 bond shortens (Supplementary Fig. 5c), reflecting a redistribution of π-bond character from Nζ = C2 to the C2=C3 double bond. In mechanistic terms, once dehydration has formed the iminium, the system readily relaxes into the enamine state, which is the reactive nucleophile poised for subsequent C–C bond formation with GALP.

### 3.2 Reaction mechanism of acceptor addition and product release in FSA

**Fig. 4:**
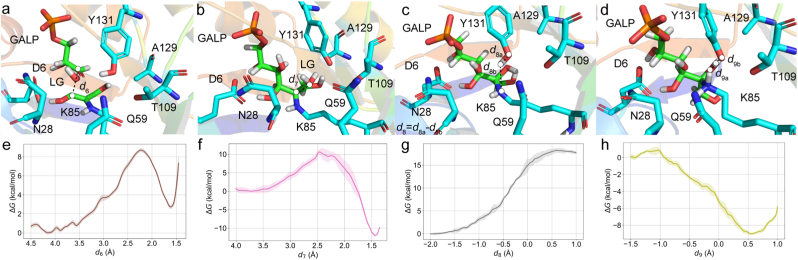
QM/MM free-energy profiles for acceptor attack and F6P release in FSA. a) Definition of the reaction coordinate *d*_6_, the distance between the nucleophilic C3 of the enamine and the aldehyde carbon of GALP (C4), describing the C–C bond formation step. b) Definition of the reaction coordinate *d*_7_, the distance between the oxygen atom of the leaving-group water (LG) and C2, describing hydrolysis on the iminium carbon to form the C2–OH bond and yield a six-carbon carbinolamine intermediate. c) Definition of the proton-transfer coordinate *d*_8_ = *d*_8a_ − *d*_8b_, reporting proton transfer from Tyr131 to the Nζ atom of Lys85, priming C–N bond cleavage during product release. d) Definition of the proton-transfer coordinate *d*_9_ = *d*_9a_ − *d*_9b_, where *d*_9a_ and *d*_9b_ are the distances between the transferring proton and the C2 hydroxyl oxygen and the phenolic oxygen of Tyr131, respectively, describing re-formation of the C2 carbonyl at the final stage of regeneration. e, f, g, h) Corresponding relevant free-energy profiles generated from QM/MM simulations for C–C bond formation (*d*_6_), hydration of the iminium at C2 (*d*_7_), Nζ protonation (*d*_8_) and C2 carbonyl restoration (*d*_9_), respectively. Data are presented as mean values ± the standard error inferred from three independent runs; shaded bands indicate the standard error.

With the donor activated as a Lys85-bound enamine, we next characterized the mechanism of C–C bond formation with GALP and the subsequent steps that lead to F6P release (Fig. 4). In the aldol step, the nucleophilic C3 of the enamine attacks the aldehyde carbon of GALP (C4, numbering in Fig. 1c), and this process was monitored by the distance coordinate *d*_6_ between these two carbons (Fig. 4a). The PMF along *d*_6_ displays a moderate barrier of 8.7 kcal/mol as the two centers approach from ∼4.5 Å to ∼2.0 Å, followed by a shallow minimum at ∼1.6 Å (Fig. 4e). This is consistent with formation of a covalent Lys85-bound hexitol-6-phosphate iminium intermediate. Importantly, the developing oxyanion formed upon nucleophilic addition is stabilized by proton donation from Tyr131 to the acceptor carbonyl oxygen (i.e., the former aldehyde oxygen of GALP), rather than to the donor C2 position. Upon C–C bond formation, the proton from Tyr131 simultaneously transfers to the alkoxide of C4 (origin aldehyde carbon, numbering in Fig. 1c, Supplementary Movie 3). We speculate that the orientation of Tyr131 may be one factor controlling the formation of fructose-6-phosphate (3R,4S) rather than tagatose-6-phosphate (3S,4S). In this intermediate, Asp6 and Asn28 maintain hydrogen-bond contacts with the donor–acceptor moiety and help pre-organize the emerging hexitol-6-phosphate scaffold.

Following C–C bond formation, the C6 iminium adduct must be hydrolyzed to reintroduce water at C2 and initiate regeneration toward product release. Notably, the leaving group (LG) generated in the preceding dehydration step remains positioned near the iminium carbon (C2) in the post-aldol intermediate, aided by the orientation of deprotonated Ty131. We described this hydration by the distance coordinate *d*_7_, defined as the distance between the oxygen atom of the LG and C2 (Fig. 4b). The PMF along *d*_7_ exhibits a barrier of 10.7 kcal/mol and an overall downhill free-energy change of 12.0 kcal/mol as the LG–C2 distance decreases from ∼3.8 Å to 1.43 Å (Fig. 4f), indicating that water addition to the iminium carbon and formation of the C2–OH bond to yield the six-carbon carbinolamine intermediate are thermodynamically favorable. This step effectively “recycles” the expelled water to reverse Schiff-base formation, thereby setting the stage for subsequent C–N bond cleavage and restoration of the C2 carbonyl during late-stage turnover.

We then examined the steps that cleave the Lys85–C2 bond and restore the carbonyl at C2 to complete product release. The breakdown of the carbinolamine was monitored by an antisymmetric distance coordinate *d*_8_ = *d*_8a_−*d*_8b_, which reports proton transfer from Tyr131 to the Nζ atom of Lys85 while the covalent linkage is still present (Fig. 4c). Along *d*_8_, the barrier is 18.2 kcal/mol, indicating that reprotonation of Lys85 Nζ by Tyr131 is a late-stage activation step that destabilizes the Lys85–C2 carbinolamine linkage and promotes C–N bond cleavage for F6P release. (Fig. 4g).

Then, proton abstraction from the C2 hydroxyl group by the Tyr131 tyrosinate was described by an antisymmetric coordinate *d*_9_ = *d*_9a_ − *d*_9b_. The *d*_9a_ was defined as the distance between the transferring proton and the C2 hydroxyl oxygen, and the *d*_9b_ was defined as the distance between transferring proton and phenolic oxygen of Tyr131, respectively (Fig. 4d). The PMF along *d*_9_ shows only a negligible barrier and an overall free-energy drop of 9.0 kcal/mol (Fig. 4h), indicating that this step is thermodynamically favorable for ketone formation at C2.

In the last step, the C2–Nζ bond (*d*_1_) gradually elongates as the C2 carbonyl is restored, facilitating dissociation of the Lys85–sugar intermediate and regeneration of the neutral Lys85 nucleophile along with fructose-6-phosphate release (Supplementary Fig. 6). Together, our results suggest that the highest barrier is associated with Nζ protonation/activation for C–N cleavage (*d*_8_), whereas the C–C bond formation itself is comparatively facile in EcoFSA under the present model. This may be the underlying mechanism that EcoFSA catalyzes the reversible aldol reaction.

### 3.3 Rational design of FSA for improved activity

To rationally enhance the catalytic performance of EcoFSA, we first focused on donor activation. Our QM/MM-derived mechanism indicates that dehydration along the carbinolamine-to-iminium conversion represents the highest barrier within donor activation. Together, these observations motivated us to prioritize active-site residues that can reorganize the local proton-transfer and water-mediated hydrogen-bond network around Lys85 and Tyr131.

Among the residues proximal to the Lys85–Tyr131 catalytic dyad, we selected Asn28, Thr109, and Ala129 for targeted diversification, because these residues reside in the immediate vicinity of the catalytic center and are structurally poised to (i) tune the orientation and acidity of Tyr131 (Thr109), (ii) stabilize the donor DHA (Asn28 and Ala129).

**Fig. 5:**
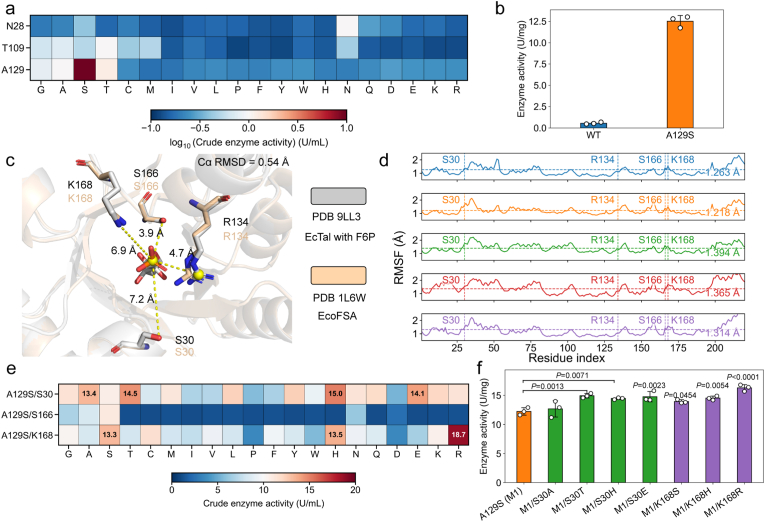
Structure-guided engineering of EcoFSA. a) Crude enzymatic activities for single-site saturation mutagenesis at Asn28, Thr109 and Ala129. b) The purified enzyme activities of wild-type EcoFSA and the variant M1. c) Structural alignment of EcoFSA (PDB 1L6W) and the EcTal–F6P complex (PDB 9LL3), highlighting a putative phosphate-recognition region. d) Heavy-atom RMSF profiles from MD simulations of an apo EcoFSA pentamer. e) The crude lysate activities for saturation mutagenesis at Ser30, Ser166 and Lys168 based on the A129S (M1). f) The purified enzyme activities of combinatorial double-site variants. For panels b and f, open circles indicate individual replicates. All data are presented from biological replicates (n = 3). Statistical analysis for panels f was performed by one-way ANOVA followed by Dunnett's post hoc test (using M1 as the control), *P* values are indicated in the panels.

Given that EcoFSA showed high thermostability, the clarified lysate was heat-treated at 60 °C for 30 min to obtain crude protein for rapid activity screening (Supplementary Fig. 7). Notably, among the Asn28/Thr109/Ala129 donor-pocket positions selected above, EcoFSA-A129S(M1) was the only single substitution that reproducibly improved the native condensation activity relative to wild type (Fig. 5a). This observation is consistent with prior reports identifying A129S as a beneficial hotspot for DHA-dependent FSA catalysis, where the Ser substitution was proposed to enable additional hydrogen bonding to stabilize DHA and Schiff-base intermediates, albeit with assay- and condition-dependent absolute kinetic/activity values across studies [26,29]. Under our discontinuous two-step dephosphorylation assay (Methods), the purified M1 exhibited an activity of 12.5 U/mg, corresponding to a 21-fold increase compared with EcoFSA-WT (0.6 U/mg) (Fig. 5b). Mechanistically, substituting Ala129 with Ser likely increases the local hydrogen-bonding capacity with the hydroxyl groups of Tyr131 and Thr109, thereby promoting the proton-transfer conformation that promotes dehydration and iminium formation. Consistently, equilibrated QM/MM MD of A129S assembly also showed that A129S can form a stable hydrogen bond and facilitate the first step of nucleophilic attack (Supplementary Fig. 8).

Then, we shifted our focus to rational engineering of the acceptor scope. Engineering the acceptor-binding region has successfully broadened the acceptor tolerance towards α-substituted and non-phosphorylated aldehydes by diminishing phosphate binding ability [30]. This suggests that the enhancement of the GALP binding may be beneficial. Although the crystal structure of EcoFSA in complex with F6P has not been resolved, a structure of transaldolase from *Enterobacter cloaca* (EcTal) in complex with F6P has recently been released (PDB 9LL3) [56]. EcTal shares 85.91% sequence identity with EcoFSA, and the structure alignment shows that the monomer Cα RMSD between EcTal and EcoFSA is only 0.54 Å over all 220 aligned residues. Previous studies have highlighted that second-shell interactions around phosphorylated substrates can disproportionately influence binding geometry and catalytic energy barrier [57,58], even when the residues do not directly contact the phosphate moiety. Accordingly, we identified four residues located within the second-shell region (≤8 Å from the phosphate group) in the putative GALP-binding site, namely Ser30, Arg134, Ser166, and Lys168 (Fig. 5c).

To probe the dynamics of the phosphate-recognition module, we performed MD simulations on the EcoFSA pentamer, which preserves native inter-subunit packing of the decameric assembly (Supplementary Movie 4). Heavy-atom RMSF across five chains shows that Ser30, Ser166, and Lys168 are consistently more flexible (Fig. 5d). Accordingly, we prioritized Ser30, Ser166, and Lys168 for further mutagenesis to assess how their conformational plasticity shapes GALP binding and turnover.

Then, saturation mutagenesis was performed at the three residues using EcoFSA-M1 as the template. Among the tested variants, seven single-site variants showed increased crude enzyme activity relative to the control (Fig. 5e). The corresponding variants were further purified, and their activities were measured using DHA and GALP as substrates. For Ser30 site, four variants of M1-S30A, M1-S30T, M1-S30H, and M1-S30E showed increased crude enzyme activity. Although mutant M1-S30A showed a significant increase in crude enzyme activity, its purified enzyme activity did not increase (Fig. 5f), likely due to increased soluble expression. For Lys168 site, mutants of M1-K168S, M1-K168H and M1-K168R showed increase in enzyme activity. Among them, the mutant M1-K168R (M2), named as EcoFSA-M2 showed the best performance with a 34% increase relative to that of EcoFSA-M1 (Fig. 5f).

### 3.4 Combinatorial mutation for increasing catalytic efficiency

**Fig. 6:**
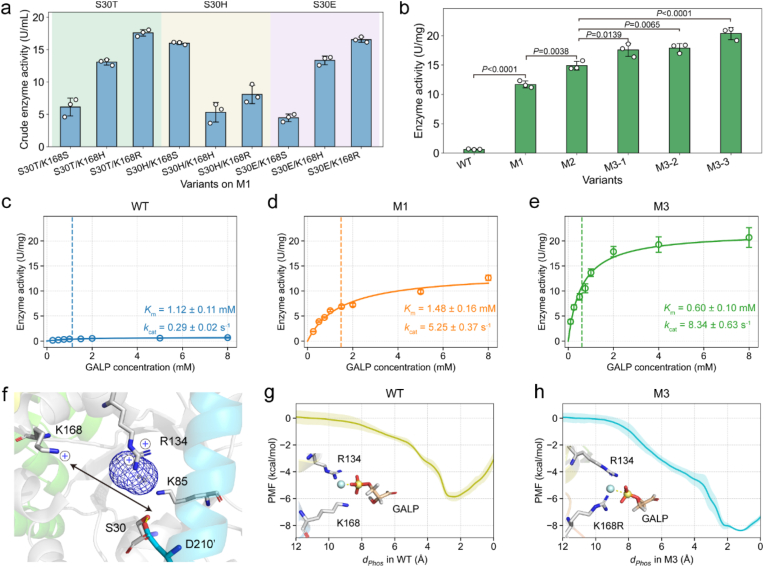
Combinatorial mutation engineering for FSA and enzymatic kinetics for its variants. a) Crude enzyme activities of nine combinatorial variants generated by combining beneficial substitutions at positions Ser30 and Lys168 on the M1 background. b) Enzyme activities of purified WT, M1 (A129S), M2 (M1-K168R), M3-1 (M1-S30H–K168S), M3-2 (M1-S30E-K168R), and M3-3 (M1-S30T-K168R). c-e) The Michaelis–Menten kinetics for GALP of WT (c), M1 (d) and M3-3 (e). f) Tunnel analysis for the EcoFSA (PDB 1L6W). The result highlighted that Ser30 is located at the TIM-barrel entrance. g, h) PMF profiles of *d*_phos_ in WT (g) and M3-3 (h). The *d*_phos_ was defined as the distance between the center of positive charge (Lys168 Nζ and Arg134 NH1/NH2) and the phosphate group of a phosphate-containing probe (PO_4_ moiety). For panel a, b, c, and d data are shown as mean ± SD and circles indicate individual replicates (n = 3, biological replicates). Statistical analysis for panels b was performed by one-way ANOVA followed by Duncan's post hoc test, *P* values are indicated in the panels.

Given the beneficial effect of mutations at sites S30 and K168, we combined these positive variants for further improving activity. To systematically explore the combinational effect, K168S, K168H and K168R were introduced into three double-site variants of M1/S30T, M1/S30H, and M1/S30E, respectively. Thus, 9 combinations were screened, and their crude enzyme activities were measured (Fig. 6a). Three triple-variants, M1-S30H–K168S (M3-1), M1-S30E-K168R (M3-2), and M1-S30T-K168R (M3-3) presented higher crude enzyme activity than with other variants. Subsequently, activity assays with purified enzymes also confirmed the screening result. Typically, the variant M3-3 exhibited the highest activity of 20.4 U/mg, presenting a 34-fold increase relative to the wild type (Fig. 6b). In addition, kinetic assays proved that mutations of S30T and K168R decreased the *K*_m_ for GALP to 0.6 mM (Fig. 6d and e).

To reveal the origin of increased affinity of M3-3 for GALP, the possible access tunnels were further analyzed in the crystal structure of EcoFSA (PDB 1L6W). Ser30 is located in the entrance of the TIM-barrel, and forms a strong hydrogen bond with Asp210 from a neighboring subunit. The S30T mutation possibly narrows the entrance and optimizes the orientation of GALP toward positively charged residues (Fig. 6f). To identify the phosphate binding ability of M3 variant, we defined a CV *d*_phos_ as the distance between the center of positive charges (NZ of Lys168 and NH1, NH2 of Arg134) and negative charge (PO4 moiety of GALP), to simulate the process of phosphate recruitment from solution. The PMF results for *d*_phos_ demonstrated that the K168R variant stabilized the phosphate binding by 2.4 kcal/mol, compared with the unbound state (Fig. 6g and h). The K168R variant is expected to provide stronger electrostatic interactions. Notably, a similar substitution at this site was also discovered in transaldolase B from *E. coli*, indicating that Arg and Lys could interchange during evolution to adapt enzyme function [17].

Taken together, for the beneficial mutant M3-3, the A129S mutation boosts the chemical competence of the donor activation, whereas K168R and S30T may enhance phosphate-assisted acceptor recruitment and charge distribution.

### 3.5 Employment of engineered FSA for one-carbon conversion to mannitol

**Fig. 7:**
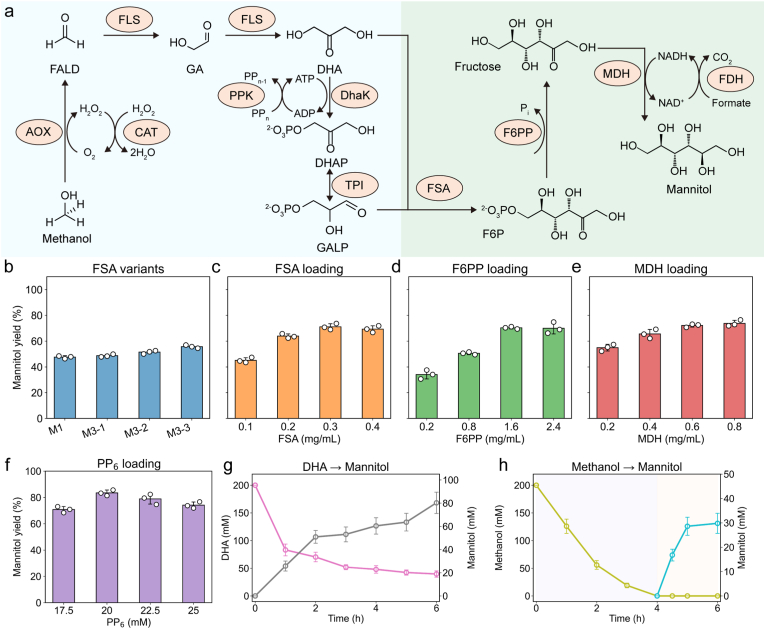
*In vitro* multi-enzyme cascade reactions for conversion of methanol to mannitol. a) Pathway design for C1-to-mannitol. The synthetic pathway could be divided into two parts, termed the C1-to-C3 and C3-to-C6 modules. b) The effect of FSA variants on mannitol production in the C3-to-C6 module. c-f) The effect of enzyme loading and polyphosphate (PP_6_) concentration on mannitol production. EcoFSA (c), F6PP (d), MDH (e), and polyphosphate (PP_6_) concentration (f). g) Time-course of DHA-to-mannitol conversion under optimized C3-to-C6 conditions with 200 mM DHA as substrate. h) The methanol-to-mannitol conversion with 200 mM methanol as substrate. For panels b–h, data are shown as mean ± SD and circles indicate individual experiments (n = 3, biological replicates).

Mannitol is a six-carbon sugar alcohol with wide applications as a low-calorie sweetener and a diuretic in the food and pharmaceutical industries. While biotransformation of fructose or starch-derived sugars to mannitol has been extensively studied, converting C1 feedstocks (e.g., CO_2_-derived methanol) into mannitol remains far less developed. Here, we designed an *in vitro* multi-enzyme cascade that couples C1 assimilation with FSA-enabled C–C bond formation to produce mannitol (Fig. 7a). Conceptually, the pathway can be divided into two modules: a C1-to-C3 module that converts methanol into DHA, and a C3-to-C6 module that converts two molecules of DHA into mannitol. Notably, the C1-to-F6P pathway followed the previously reported ACSP chemoenzymatic cascades for C1/CO_2_-to-sugar conversion [12]; here, we connected two downstream reactions F6PP-catalyzed dephosphorylation and MDH-catalyzed reduction reactions toward mannitol formation from F6P. In the C1-to-C3 module, alcohol oxidase (AOX) catalyzes the oxidation of methanol to formaldehyde. Although NAD-dependent methanol dehydrogenases can also convert methanol to formaldehyde with concomitant NADH generation, their catalytic efficiencies are relatively low and may limit pathway flux [59,60]. Therefore, AOX was used for methanol oxidation in the C1-to-C3 module, while catalase was included to remove the hydrogen peroxide generated during AOX catalysis. To further validate the quality of the AOX preparation used in the upstream oxidation module, we quantified its FAD loading spectrometrically. The measured FAD content closely approached the theoretical value expected for full occupancy, indicating that the AOX used in this study displayed high, near-stoichiometric FAD loading (Supplementary Figs. 9 and 10; Supplementary Table S1). Subsequently, a formolase mutant (FLS-M3) converts formaldehyde into DHA. In the C3-to-C6 module, DHA is phosphorylated by DhaK to DHAP, ATP is regenerated from polyphosphate by PPK, TPI establishes the DHAP/GALP isomerization; EcoFSA then condenses DHA and GALP to yield F6P, which is subsequently dephosphorylated by F6PP to fructose, and finally reduced to mannitol by MDH coupled with NADH regenerated by FDH using formate as the reductant (Fig. 7a). Overall, the synthesis of one mannitol molecule via this *in vitro* route consumes 6 equivalents of methanol, 6 equivalents of molecular oxygen, 1 equivalent of formate and 1 phosphate-bond equivalent, while generating 1 molecule of inorganic phosphate and 1 CO_2_ as byproducts.

Given that the upstream methanol-to-F6P module has been well established in ACSP [12], we focused the enzyme-loading optimization on the DHA-to-mannitol module, where the pathway is newly extended toward mannitol and the performance of engineered EcoFSA variants on product formation could be measured. To demonstrate the feasibility of *in vitro* enzyme cascade for mannitol production, an initial test using DHA as the substrate was performed for the C3-to-C6 module. The enzymes PpDhaK, EcTPI, EcoFSA-M1, F6PP3, LpMDH, MtPPK and MvFDH were assembled in this synthetic module. The initial reaction employed 30 mM DHA and 20 mM formate. This designed system produced 8.25 mM mannitol with a conversion yield of 55% (Fig. 7b). When introducing other beneficial mutants of FSA such as EcoFSA-M3-1, EcoFSA-M3-2, EcoFSA-M3-3 into the reaction system, the mannitol production slightly increased (Fig. 7b). We then optimized the enzyme loadings of EcoFSA-M3-3, F6PP, and MDH, and the polyphosphate concentration, resulted in a high conversion yield of 80.3% (Fig. 7c, d, 7e, and 7f). Guided by the optimized enzyme combinations, the initial substrate concentrations were elevated to 200 mM DHA and 150 mM formate (Fig. 7g). Under this condition, mannitol production increased to 80 mM with the similar conversion yield. Beneficially, our designed EcoFSA-M3 decreased the enzyme amount in reaction system (0.15 mg/mL for 200 mM DHA) by 3-fold compared with our previous study (0.5 mg/mL for 200 mM DHA) [31].

Finally, we integrated the C1-to-C3 and C3-to-C6 modules into a one-pot, two-step cascade reaction starting from methanol. This design minimizes potential cross-inhibition between modules, as methanol oxidation via AOX generates formaldehyde and H_2_O_2_ that can compromise downstream enzyme performance [61,62]. In the first stage, a combination of AOX, catalase, and the formolase mutant successfully converted methanol into DHA. After ∼4 h, the stage 1 reaction converted methanol to DHA, and stage 2 was initiated by adding downstream enzymes and cofactors for the C3-to-C6 module. Mannitol accumulated to 29.8 mM within the following 2 h with a conversion yield of 88.8% (Fig. 7h).

## 4 Conclusions

In this study, we elucidated the catalytic cycle of the FSA-mediated aldol reaction between DHA and GALP using QM/MM simulations in combination with enhanced sampling, thereby providing a mechanistic interpretation of donor activation, acceptor addition, and product release. Based on the reaction mechanism, we rationally engineered FSA to improve its catalytic performance and obtained several beneficial mutants possessing a 34-fold increase in catalytic efficiency relative to wild-type FSA. Finally, we integrated the engineered FSA into a designed *in vitro* biotransformation platform for one-carbon conversion, demonstrating its utility in constructing efficient synthetic routes toward mannitol. Collectively, this work establishes a mechanistically grounded framework for aldolase engineering and supports the development of scalable synthetic pathways for one-carbon valorization.

## CRediT authorship contribution statement

**Dandan Wang:** Validation, Investigation, Data curation. **Qianzhen Dong:** Writing – original draft, Visualization, Formal analysis, Data curation. **Peng Chen:** Validation, Methodology. **Yan Zeng:** Validation, Resources, Data curation. **Yinlu Liu:** Investigation, Formal analysis, Data curation. **Yuanxia Sun:** Writing – review & editing, Resources, Project administration. **Jianxin Tan:** Writing – review & editing, Resources, Project administration, Conceptualization. **Jiangang Yang:** Writing – review & editing, Writing – original draft, Funding acquisition, Formal analysis, Conceptualization.

## Declaration of competing interest

The authors declare that they have no known competing financial interests or personal relationships that could have appeared to influence the work reported in this paper.
