# Supplementary material for: Engineering of fructose-6-phosphate aldolase for one-carbon conversion to mannitol in a designed biotransformation system
Source: Synth Syst Biotechnol. 2026 Jun 5;14:447–58. doi: 10.1016/j.synbio.2026.04.009 (PMC13259622; doi:10.1016/j.synbio.2026.04.009)
Supplement: Multimedia component 1 [file mmc1.docx]

Supplementary Information

**Engineering of fructose-6-phosphate aldolase for one-carbon conversion to mannitol in a designed biotransformation system**

Dandan Wang^1,2†^, Qianzhen Dong^2,3†^, Peng Chen^2,3^, Yan Zeng^2^, Yinlu Liu^2^, Yuanxia Sun^2,3*^, Jianxin Tan^1*^, Jiangang Yang^2,3*^

^1^ College of Food Science and Technology, Hebei Agricultural University, Baoding, 071001, China

^2^Tianjin Institute of Industrial Biotechnology, Chinese Academy of Sciences, Tianjin 300308, China.

^3^Key Laboratory of Engineering Biology for Low-carbon Manufacturing, Tianjin 300308, China.

^†^ These authors contributed equally to this work

*Correspondence: sun_yx@tib.cas.cn; [jianxintan@sina.com](mailto:jianxintan@sina.com); yang_jg1@tib.cas.cn


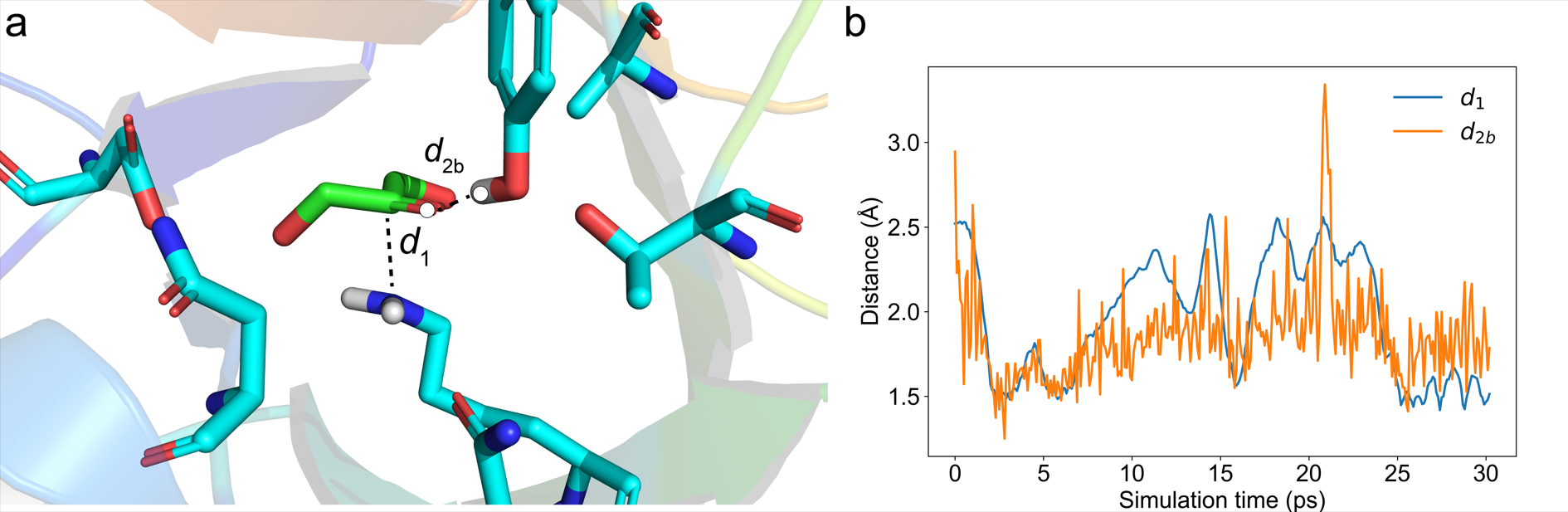


**Supplementary Figure 1** | Early pre-organization during nucleophilic attack on DHA.

(a) Representative snapshot of the first nucleophilic attack showing that the former carbonyl oxyanion of DHA is positioned to strongly attract the phenolic proton of Tyr131, consistent with early pre-organization for the ensuing proton-transfer step. (b) Time evolution of distances illustrating the tight Tyr131–oxyanion proximity concomitant with formation of the Lys85–C2 adduct.


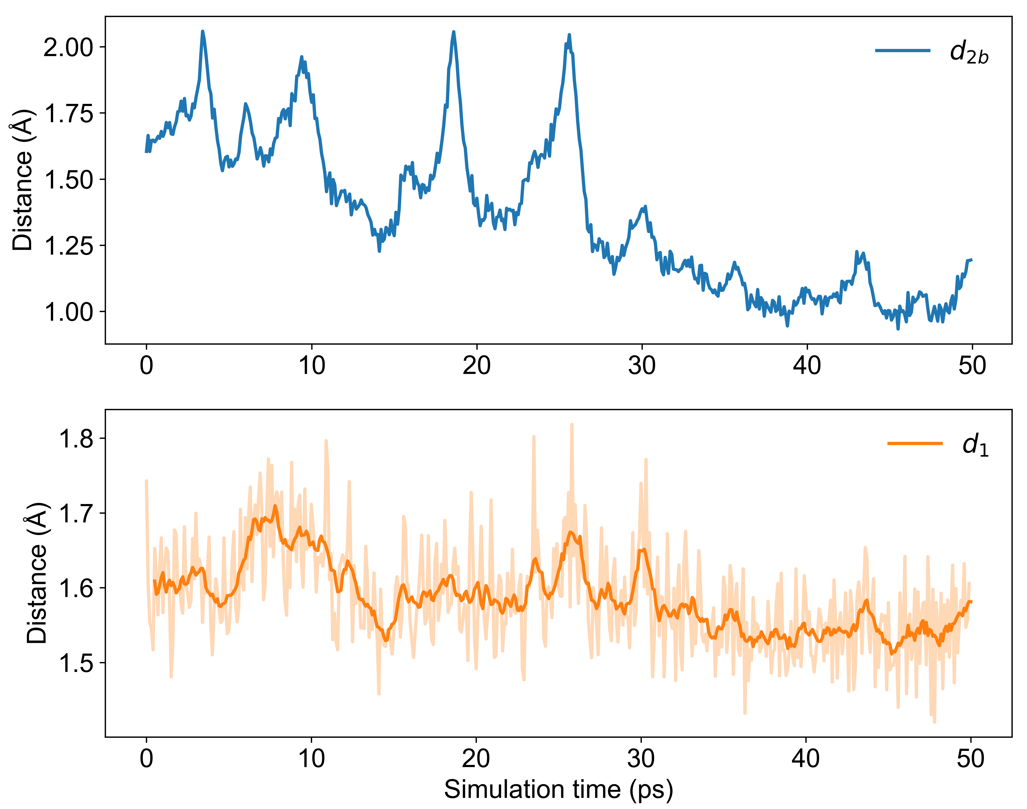


**Supplementary Figure 2** | Distance evolution during Tyr131-to-donor proton transfer (*d*_2_).

Representative time series of *d*_1_ and *d*_2b_ showing that the Nζ–C2 bond is further tightened during the proton-transfer process (*d*_2b_).


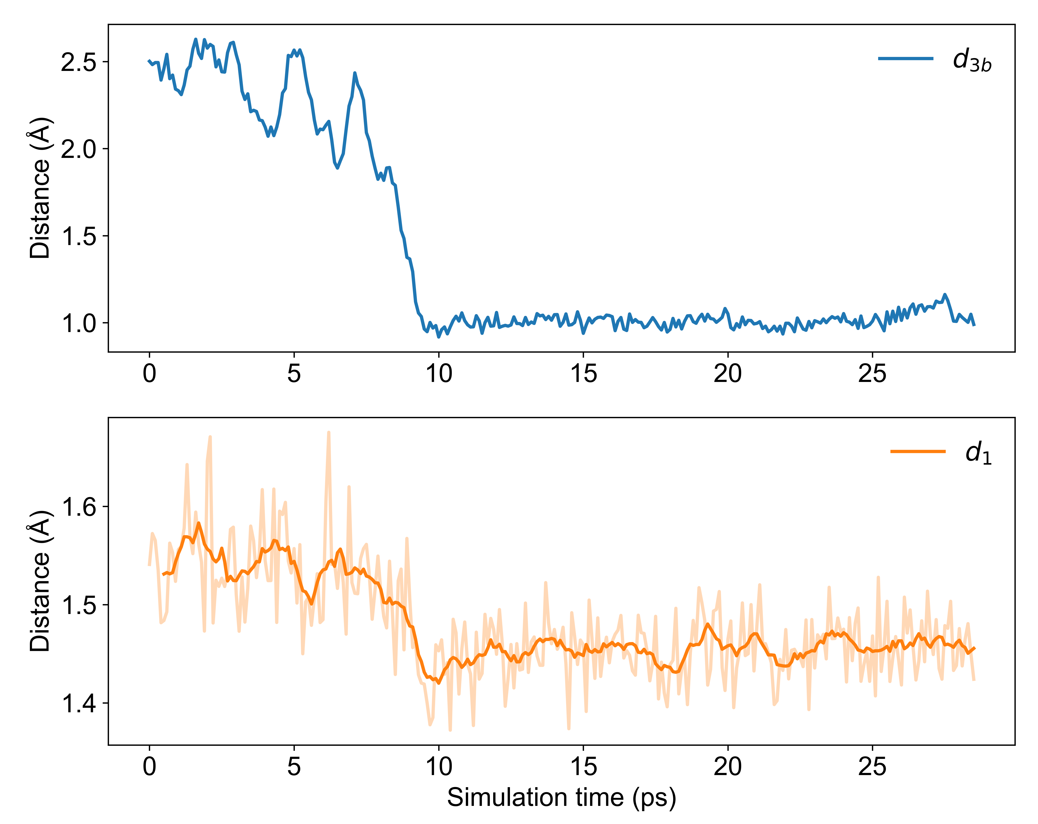


**Supplementary Figure 3** | Distance evolution during carbinolamine formation (*d*_3_).

Representative time series of *d*_1_ and *d*_3b_ show that the Nζ–C2 bond continues to shorten with the transferable proton becomes closer to Tyr131 (*d*_3b_) during the *d*_3_ process.


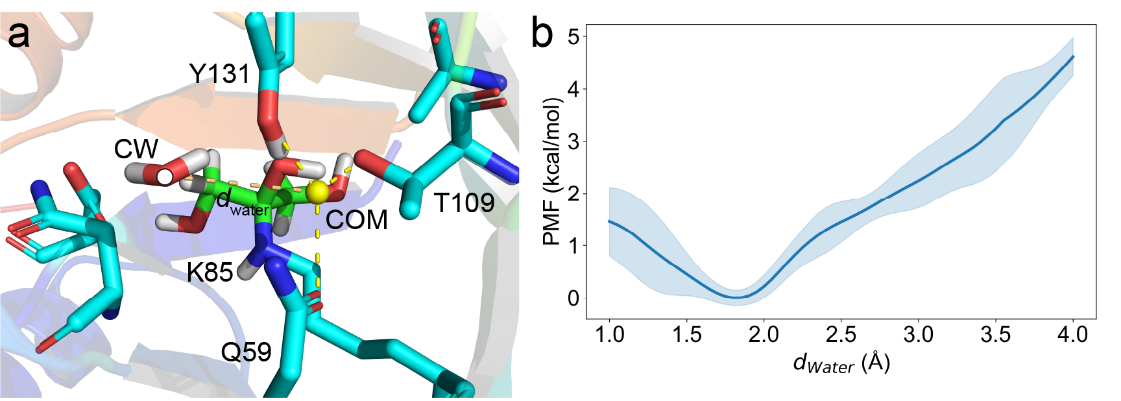


**Supplementary Figure 4** | A catalytic-water binding site organized by the Q59–T109–Y131 triad.

(a) Definition of *d*_water_ as the distance between the catalytic water oxygen and the center-of-mass (COM) of the sidechain oxygen atoms of Gln59, Thr109, and Tyr131. (b) PMF along *d*_water_ shows a pronounced minimum at ~1.8 Å, indicating a well-defined, strongly bound catalytic-water site in the Q59–T109–Y131 pocket.


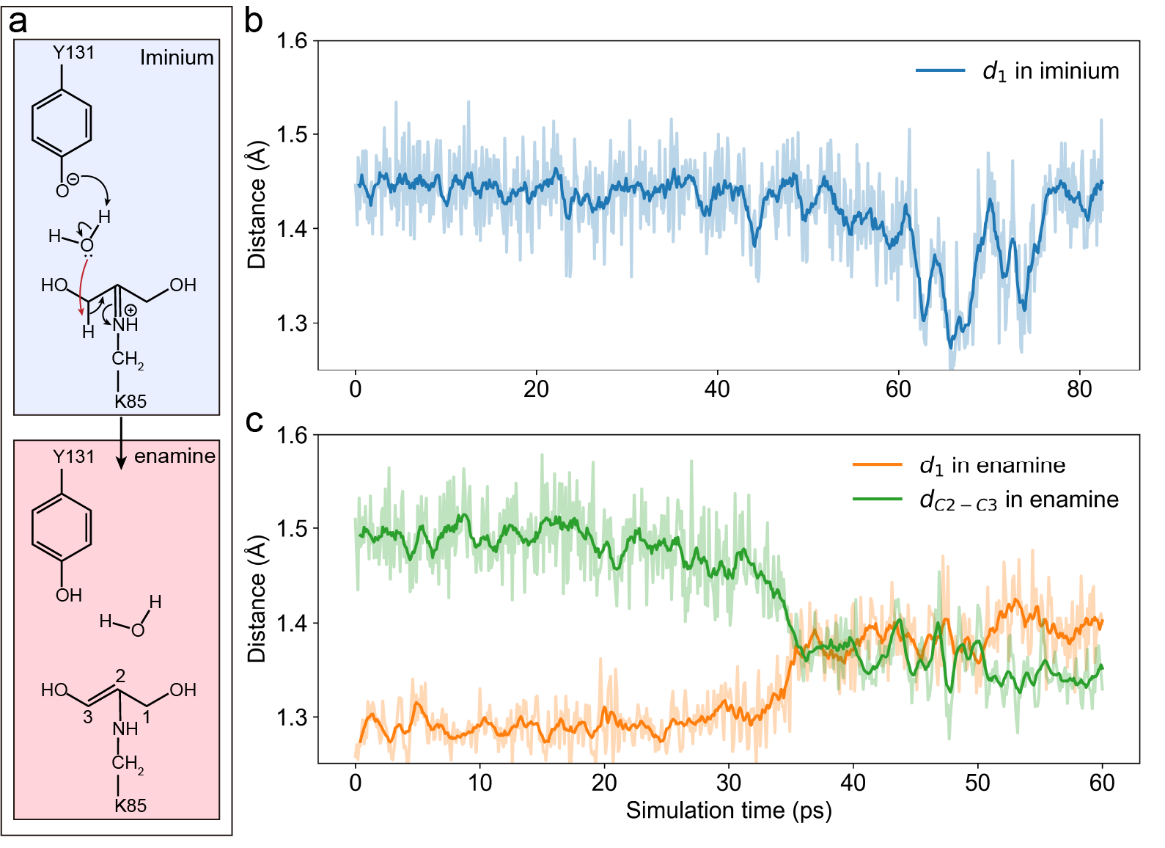


**Supplementary Figure 5** | Bond-length signatures of iminium and enamine formation.

(a) Schematic of donor activation highlighting iminium formation and α-deprotonation to the enamine. (b) Representative time evolution of *d*_1_ showing shortening of the Lys85 Nζ–C2 distance from single-bond-like values (carbinolamine region) to double-bond-like values (iminium region). (c) Representative bond-length evolution during enamine formation. It shows that *d*_1_ increases while the adjacent C2–C3 bond shortens, consistent with redistribution of π-bond character from Nζ=C2 (iminium) to C2=C3 (enamine).


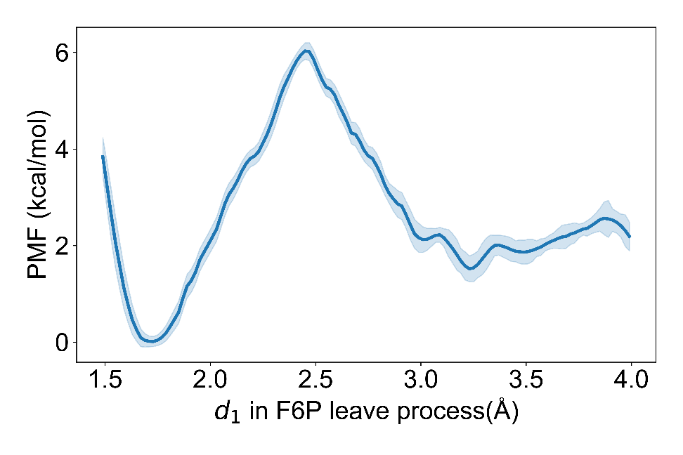


**Supplementary Figure 6** | Free-energy profile for product release via Lys85–C2 bond dissociation. The potential of mean force (PMF) is plotted along *d*_1_, defined as the distance between Lys85 Nζ and C2 of the covalent sugar adduct, which monitors cleavage of the Lys85–sugar linkage during F6P release and regeneration of the catalytic Lys85. The profile shows a modest barrier (~6 kcal/mol) for F6P release.


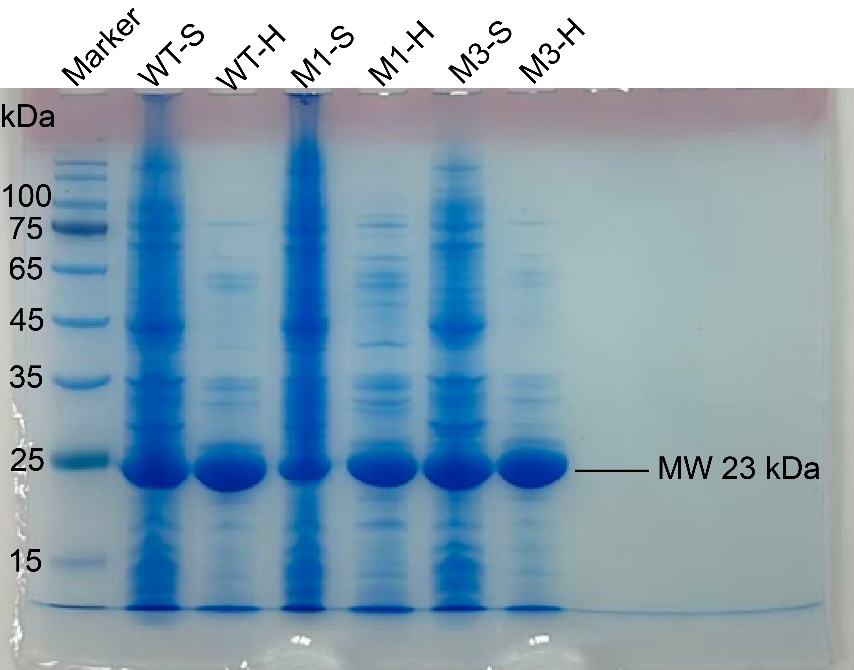


**Supplementary Figure 7** | SDS–PAGE assessment of crude EcoFSA preparations for rapid screening. SDS–PAGE analysis of crude enzyme preparations before and after heat treatment used for rapid activity screening of EcoFSA-WT and variants. Lane assignments: Marker; S, soluble lysate; H, supernatant after heat treatment.


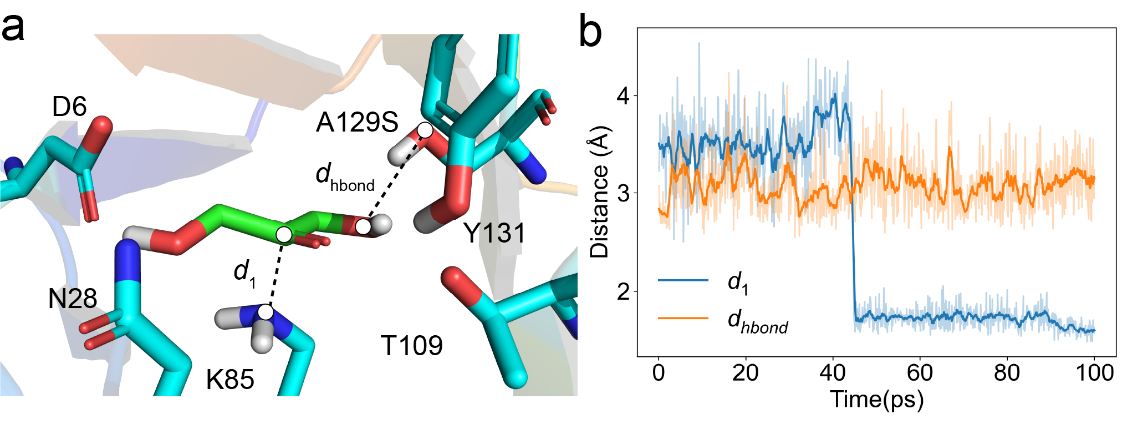


**Supplementary Figure 8** | A129S forms a stabilizing hydrogen bond that assists nucleophilic attack on DHA. (a) Representative snapshot illustrating the hydrogen bond formed by Ser129 and DHA. (b) Representative distance time series showing a persistent Ser129-mediated hydrogen bond and a spontaneous progression of the nucleophilic-attack event in the A129S assembly.


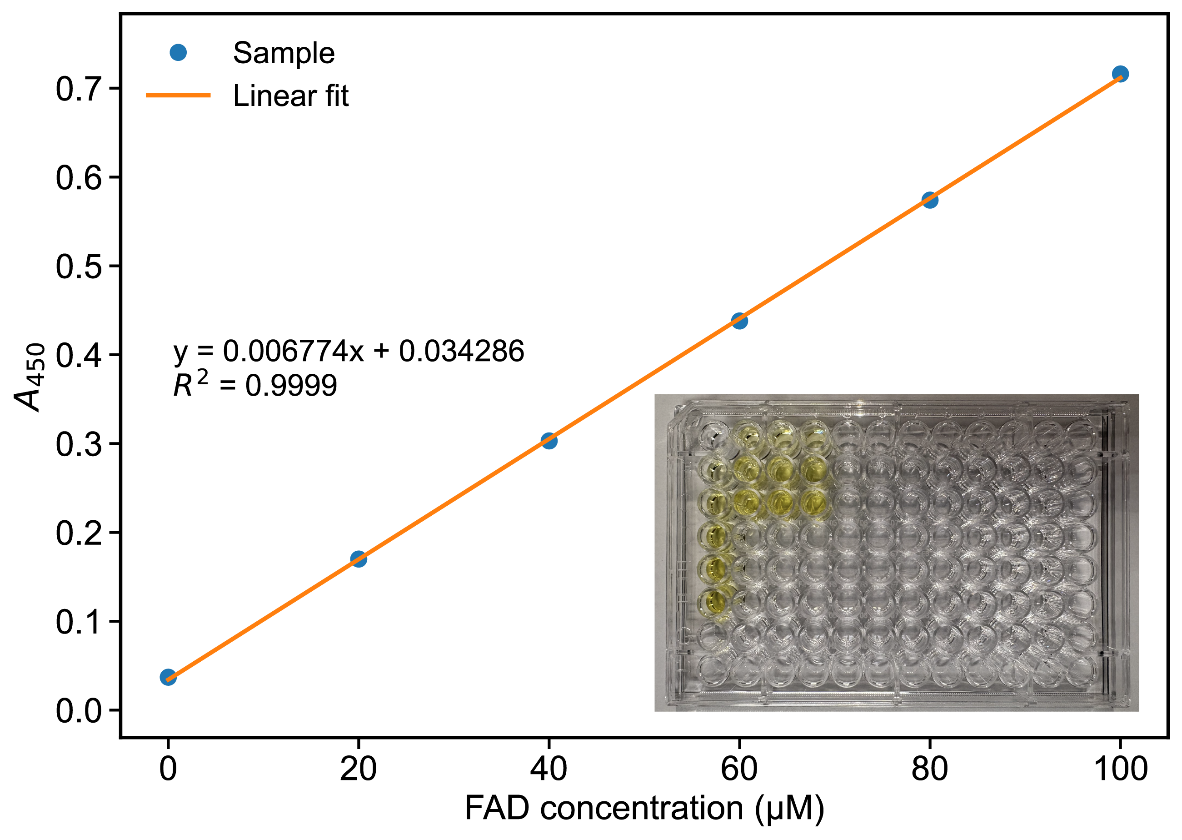

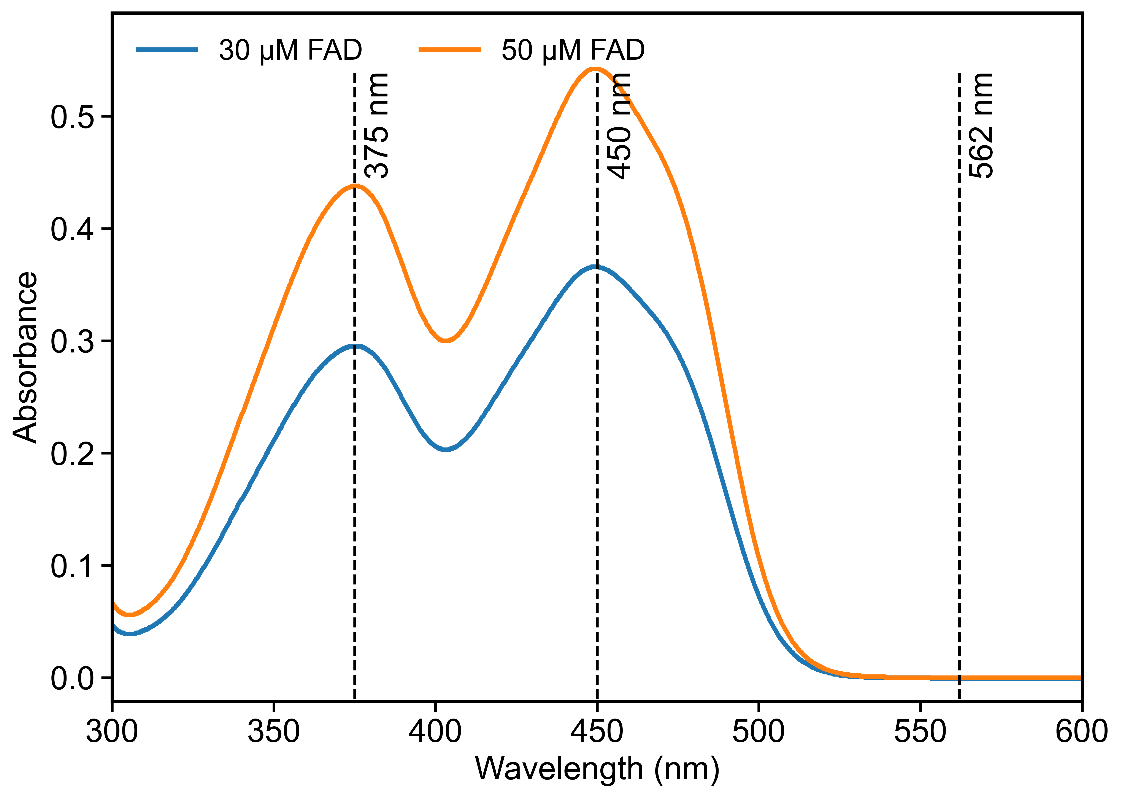
**Supplementary Figure 9** | UV–visible absorption spectra of free FAD. UV–visible absorption spectra of free FAD at 30 and 50 μM recorded from 300 to 600 nm in a quartz cuvette. The spectra showed the characteristic flavin absorption bands around 375 and 450 nm. No appreciable absorbance was observed at 562 nm, supporting the use of A450 for flavin quantification and the BCA assay (A562) for AOX protein determination.

**Supplementary Figure 10** Microplate-based standard curve for FAD quantification. Standard curve for FAD quantification at 450 nm in a 96-well microplate using 0, 20, 40, 60, 80, and 100 μM FAD (200 μL per well).

**Supplementary Table 1** Quantification of AOX flavin loading. Measured flavin concentrations released from AOX samples at different protein concentrations and calculation of flavin occupancy based on the expected stoichiometry of one FAD per 74-kDa AOX monomer.

| **AOX (mg/mL)** | **A_450_** | **Measured FAD (µM)** | **Theoretical occupancy (µM)** | **Occupancy (%)** |
| --- | --- | --- | --- | --- |
| 2 | 0.190±0.009 | 22.95±1.37 | 27.06 | 84.8 |
| 4 | 0.359±0.012 | 47.70±1.80 | 54.13 | 88.1 |
| 6 | 0.528±0.013 | 72.60±1.84 | 81.19 | 89.4 |

We also revised the manuscript as follows:
